# The Role of Biomarkers in the Early Diagnosis of Gastric Cancer: A Study on CCR5, CCL5, PDGF, and EphA7

**DOI:** 10.3390/cimb46090632

**Published:** 2024-09-23

**Authors:** Süleyman Bademler, Berkay Kılıç, Muhammed Üçüncü, Alisan Zirtiloglu, Burak İlhan

**Affiliations:** 1 Department of Surgery, Oncology Institute, Istanbul University, 34093 Istanbul, Turkey; 2 Department of Health Science Institute, Istanbul Gelisim University, 34310 Istanbul, Turkey; 3 Department of Medical Oncology, Bakirkoy Dr. Sadi Konuk Training and Research Hospital, University of Health Sciences, 34147 Istanbul, Turkey; 4 Department of Surgery, Istanbul Faculty of Medicine, Istanbul University, 34093 Istanbul, Turkey

**Keywords:** chemokine, cytokine, gastric cancer, CCR5, CCL5, EphA7, PDGF

## Abstract

Despite the use of screening programs, gastric cancer (GC) diagnosis may only be possible at an advanced stage. In this study, we examined the serum levels of C-C chemokine receptor type 5 (CCR5), C-C motif chemokine ligand 5 (CCL5), platelet-derived growth factor (PDGF), and EphrinA7 (EphA7) in patients with gastric carcinoma and healthy controls to investigate the significance and usability of these potential biomarkers in the early diagnosis of GC. The study enrolled 69 GC patients and 40 healthy individuals. CCR5, CCL5, PDGF-BB, and EphA7 levels, which have been identified in the carcinogenesis of many cancers, were measured in the blood samples using the ELISA method. CCR5, CCL5, PDGF-BB, and EphA7 were all correlated with GC diagnosis (CCR5, *p* < 0.001, r = −0.449; CCL5, *p* = 0.014, r = −0.234; PDGF-BB, *p* < 0.001, r = −0.700; EPHA7, *p* < 0.001, r = −0.617). The serum CCR5, EphA7, and especially the PDGF-BB levels of the patients diagnosed with GC were discovered to be significantly higher compared to the healthy controls. PDGF-BB had the highest positive and negative predictive values when evaluated in ROC analysis to determine its diagnostic significance (cut-off value: 59.8 ng/L; AUC: 0.92 (0.87–0.97)). As far as we know, this is the first study to investigate the potential connection between GC and these four biomarkers. The fact that serum CCR5, CCL5, EphA7, and especially PDGF-BB levels in the patient group were significantly higher compared to healthy controls indicates that they can be used with high accuracy in the early diagnosis of GC. In addition, the levels of CCR5, PDGF-BB, and EphA7 can be used as important indicators to predict the biological behavior and prognosis of GC.

## 1. Introduction

Gastric cancer (GC) is currently the fifth most common cancer diagnosed and the cause of cancer-related mortality worldwide. Cancer-related mortality and survival rates are significantly worse in advanced disease and much better in the setting of early diagnosis. GC develops on a multifactorial basis. These include factors that cannot be modified, such as increasing age, male gender, and race/ethnicity, as well as those can be controlled, such as H. pylori infection, dietary modification, tobacco and alcohol use, and exercise. Sex makes a difference in the incidence of GC, which is almost twice as high in males. That may be explained by the protective role of sex steroid hormones in GC pathogenesis and the fact that males tend to have a higher risk of Helicobacter pylori infection. Although less common, EBV infection, gastroesophageal reflux disease, gastric ulcer, and previous gastric surgery may also have effects [1,2,3,4]. Furthermore, CDH1 gene mutation, IL-17 and IL-10 polymorphisms, Lynch syndrome, FAP (familial adenomatous polyposis), and GAPPS (gastric adenocarcinoma and proximal polyposis of the stomach) are all cancer-predisposing genetic disorders that may lead to GC. The prognosis of GC is often grave and requires a close follow-up of the patient and meticulous treatment techniques like radical surgery, chemotherapy, and sometimes radiotherapy [5]. Therefore, it is even more critical to determine early detection strategies for precise postoperative interventions. Since 1909, research has been ongoing for the early diagnosis of GC. However, there are still no current widely accepted methods, possible biomarkers for early diagnosis, or patient-specific risk factor analyses available for daily practice except for endoscopic screening programs [6,7]. Therefore, since many biomarkers have been identified in the development of different cancers, the importance of some biomarkers that can provide or support early diagnosis and that can be analyzed in tissue samples or especially in serum has increased even more.

Cancer is a complex structure that develops through dynamic interactions between cancer cells and the tumor microenvironment (TME). The TME has an effect in promoting cancer cell proliferation. This promoting effect results in the emergence of products such as chemokines, cytokines, and various growth factors to actively recruit diverse cell types to the TME of cancer cells. These cells in the TME also promote cancer cell invasion, the activation of angiogenesis, reductions in the immune response, and thus, tumor aggressiveness [8,9]. So far, numerous cytokines and chemokines that regulate chemotaxis between cells have been identified. Some of them are even thought to be formed at the beginning of the process, leading to cancer. Chemokines are small cytokines that promote the interaction of various cell types. Different cell types in the TME, including leukocytes, endothelial cells, fibroblasts, and cancer cells, produce these chemokines [10].

C-C chemokine ligand 5 (CCL5), also known as RANTES (Regulated upon Activation, Normal T cell Expressed, and Secreted), is a potent chemoattractant for lymphocytes, NK cells, eosinophils, and basophils. CCL5 is a product of cancer structure or stromal elements at the tumor niche and microenvironment. CCL5 activity is mediated mainly by binding with high affinity to C-C chemokine receptor type 5 (CCR5). CCR5 is a chemokine receptor that mediates the physiological functions of immune cells and promotes inflammatory activity by inducing cell clustering at the site of inflammation. But also, CCR5 expression may be induced selectively during malignant transformation by promoting cell proliferation, angiogenesis, and metastasis as a protumorigenic effect. Various immune cells, stromal cells, and central nervous system cells like neurons, astrocytes, and microglia may express CCR5. In the TME, CCL5 or CCR5 overexpression is reported in many cancers such as head and neck, breast, prostate, pancreatic, esophageal, gastric, colorectal, and hematological malignancies [11,12,13,14].

Platelet-derived growth factor-BB (PDGF-BB) protein is a homogenous disulfide-bond dimeric ligand that binds to PDGF receptors. It plays an essential role in carcinogenesis. Among various isoforms of these proteins, PDGF-BB is effective in cell transformation and the growth and progression of tumors [15,16].

The Eph receptors are members of the tyrosine kinase receptor family and are mainly separated into two main groups, EphA and B, based on their ligands and sequences. After the foundation of the down-regulation of Ephrin type-A receptor 7 (EphA7) in gastric cell lines, Wang et al. reported that EphA7 is expressed in gastric carcinoma specimens. They claimed that EphA7 might have a role in the pathogenesis of GC [17,18]. EphA7 gene mutations were also more frequent in lung cancer specimens [19]. Other Eph receptors and ephrins are commonly expressed in tumors like esophageal, hepatic, breast, pancreatic cancer, or melanoma [20,21].

Although these four biomarkers, together or alone, have been reported in the literature to be associated with the development or progression of GC, limited data have questioned the importance of these proteins for the early detection of GC. In our study, we investigated the potential of serum levels of these molecules in terms of the diagnosis of GC.

## 2. Materials and Methods

### 2.1. Patients

Our study included 109 persons examined in our tertiary-care health center between February 2015 and June 2018. Among these, 69 persons with GC were in the patient group. The other 40 persons who were matched regarding health-related variables such as age or gender, without any finding of cancer but presented for routine screening, were in the control group. In the patient group, patients with resectable tumors obtained blood samples before surgery. Patients with advanced cancer who will receive primarily oncologic treatment obtained a blood sample before their first chemotherapy regimen. Patients who had previously received treatment at another center, patients with a history of previous cancer, and patients who did not consent to participate in the study were excluded from the study. Patients included in the research but without sufficient follow-up were later excluded from the study. Biomarker levels were not used to differentiate or compare early and late stages of cancer. The histological type of all cancers was adenocarcinoma. Long-term follow-ups of patients kept going with the aim of sharing long-term survival data. Informed consent was obtained from all patients, and the study was reviewed and approved by the Ethics Committee of Istanbul University.

### 2.2. Determination of Serum Levels

Identified molecule levels in the blood samples were measured using the ELISA kit (Sunred Biological Technology Co., Ltd., Shanghai, China). In this measurement, the antibody-coated wells contained a volume of 40 μL of serum and 10 μL of CCR5 antibody. At the same time, the other wells contained a volume of 50 μL of standard preparation. Subsequently, 50 μL streptavidin–HRP was added to microwells. For the formation of the antigen–antibody complex, the wells were incubated for 60 min at 37 °C. A volume of 50 μL of chromogenic reagents A and B was added before incubation for 10 min at 37 °C. The absorbance and concentration outcomes were obtained using a microplate reader (ChroMate 4300 Microplate Reader, Palm City, FL, USA).

### 2.3. Statistical Analysis

All statistical analyses were performed using the Social Sciences program, version SPSS v22 (IBM SPSS, Chicago, IL, USA). The Kolmogorov–Smirnov test investigated continuous variables for normal distribution. The Spearman correlation test determined relationships between two non-parametric continuous variables. The Mann–Whitney-U test was used to assess comparisons between clinical and laboratory parameters. ROC (receiver operator characteristics curve) analysis evaluated the diagnostic usability of the parameters. The connection between the levels of the parameters and survival times was measured using means of Kaplan–Meier curves.

## 3. Results

Between February 2011 and December 2018, 69 GC patients enrolled in the study. In the group of 69 GC patients, there were 55 (79.7%) males and 14 (20.2%) females (Table 1). Twenty-six (65%) males and fourteen (15%) females consisted of the controls. The median age of the GC patients was 61 ± 11 years, and the controls were 62 ± 7 years. There was no difference between the groups regarding the patient’s age, sex, and BMI (*p* > 0.05 for all comparisons). CCR5, CCL5, PDGF-BB, and EPHA7 were all correlated with GC diagnosis (CCR5, *p* < 0.001, r = −0.449; CCL5, *p* = 0.014, r = −0.234; PDGF-BB, *p* < 0.001, r = −0.700; EphA7, *p* < 0.001, r = −0.617).

The mean levels of CCR5 (64.8 vs. 24.8 pg/mL), PDFG-BB (272.4 vs. 16.6 ng/L), and EphA7 (8.6 vs. 1.5 ng/mL) were higher in patients than those the controls (*p* < 0.001 for each comparison); however, no difference was found between the groups regarding the median CCL5 levels (*p* = 0.141) (Table 2).

**Figure 1:**
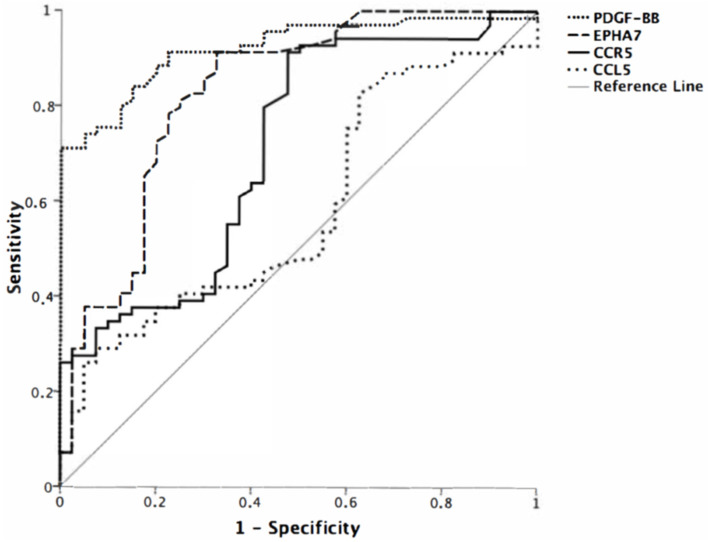
The receiver operating characteristic (ROC) analysis for serum *CCR5*, *CCL5*, *PDGF-BB*, and *EphA7* levels.

**Figure 2:**
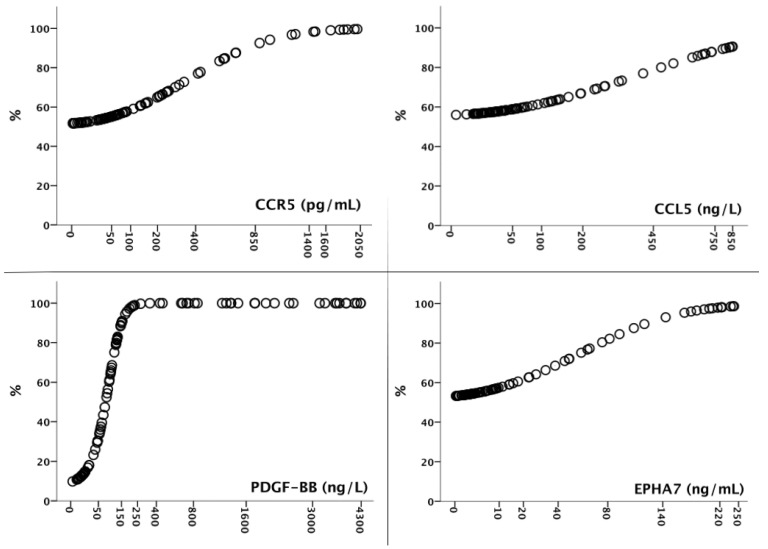
Gastric cancer probabilities (%) for serum *CCR5*, *CCL5*, *PDGF-BB*, and *EphA7* levels.

PDGF-BB had the highest positive and negative predictive values when evaluated in ROC analysis to determine its diagnostic significance (Figure 1, Table 3). The remarkable data were that the PDFG-BB level above 250 ng/mL meant that cancer probability reached 100%. GC probabilities of the molecule levels are shown in Figure 2. There was no correlation between molecule levels and the patient’s age and sex, tumor location, grade, and stage of the disease (*p* > 0.05 for all comparisons).

**Figure 3:**
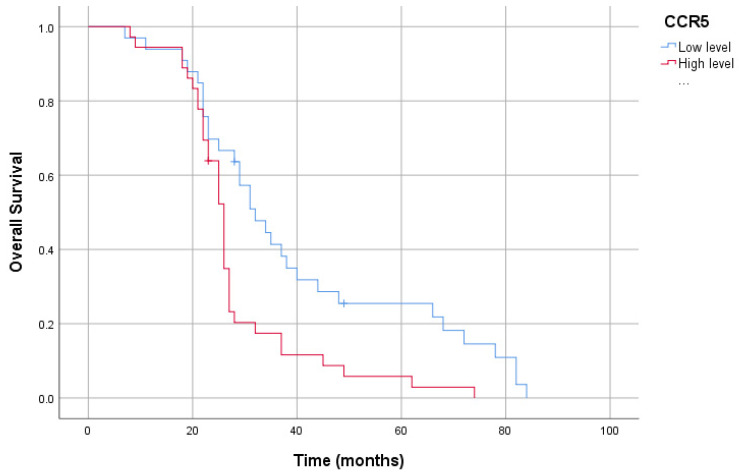
Kaplan–Meier curve of overall survival for serum *CCR5* levels.

**Figure 4:**
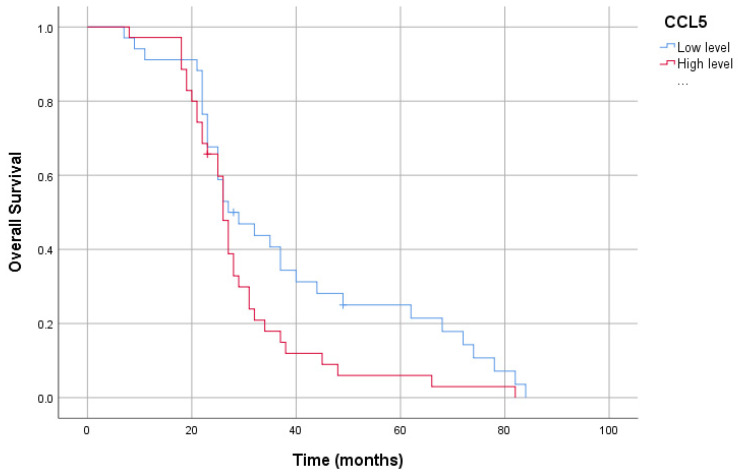
Kaplan–Meier curve of overall survival for serum *CCL5* levels.

**Figure 5:**
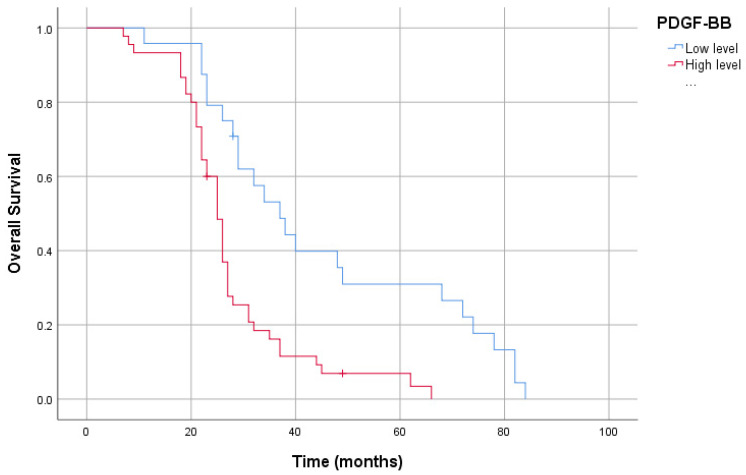
Kaplan–Meier curve of overall survival for serum *PDGF-BB* levels.

**Figure 6:**
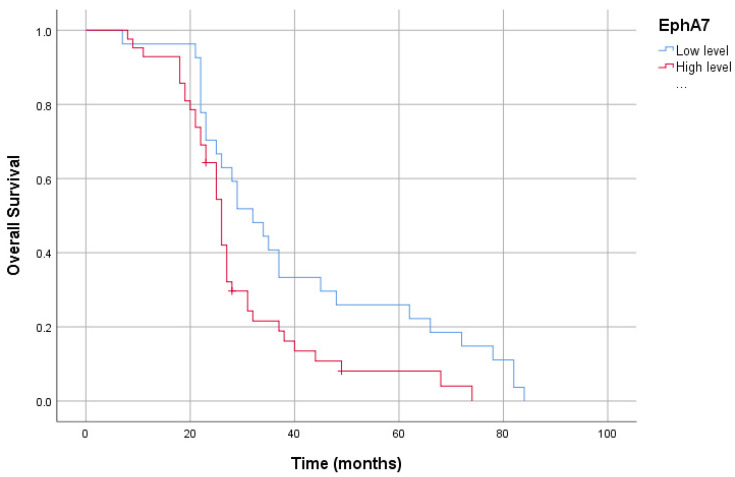
Kaplan–Meier curve of overall survival for serum *EphA7* levels.

In our study group, the patients’ 5-year survival was 13.0%. High plasma CCR5 levels in patients resulted in a shorter overall survival than low levels (40.26 ± 4.08 months vs. 27.98 ± 2.18 months, *p* < 0.05). Furthermore, high-plasma-PDGF-BB-level patients had reduced overall survival compared to low-plasma-PDGF-BB-level patients (45.24 ± 4.90 months vs. 27.59 ± 1.91 months, *p* < 0.05). Likewise, patients with high plasma EphA7 levels had reduced overall survival compared to those with low plasma EphA7 levels (40.48 ± 4.38 months vs. 29.42 ± 2.36 months, *p* < 0.05). However, there was no association between CCL5 levels and survival (38.60 ± 3.99 months vs. 29.36 ± 2.39 months, *p* > 0.05) (Figure 3, Figure 4, Figure 5 and Figure 6).

## 4. Discussion

Early diagnosis plays a vital role in reducing mortality and in better survival for all cancers. Over time, numerous diagnostic markers in cancers have been investigated, among which, several chemokines have matched the development of various cancers and their metastases [22]. The current debate is whether these proteins are appropriate as predictive agents for early diagnosis. This research aimed to assess serum CCR5, CCL5, PDGF-BB, and EphA7 levels in GC patients and age- and sex-matched healthy controls. In light of the literature, this is the first known serum study investigating these molecules in GC patients.

The CCL5/CCR5 chemokine axis, shown to be associated with various cancer types, activates tumor progression through different pathways, such as increasing tumor growth and promoting extracellular matrix remodeling, migration, invasion, and angiogenesis [23,24]. CCL5, a ligand of CCR5, is a product of cancer itself and its microenvironment, using the mTOR pathway through the regulation of cyclin D1, c-Myc, and Dad-1 expression to enhance tumor growth [25]. CCL5 has been reported to trigger invasion by increasing the release of matrix metalloproteinases 2 and 9 in prostate cancer and cancer cell motility and invasion by being secreted by mesenchymal stem cells in the TME in breast cancer [26,27].

The CCL5/CCR5 axis has also been effective in GC progression [28,29]. CCL5 expression by CD4+ tumor-associated lymphocytes in the TME and GC cells causes increased CCL5 release and enhances GC cell line growth [30]. CCR5 expression occurs via GC cell lines. Conversion to high metastatic potential through this chain of cascading mechanisms increases the expression of CCL5 in peripheral blood mononuclear cells and enhances the GC cell invasion potential [31]. Wang et al. showed that increased CCL5 levels correlated with advanced disease and worse survival in patients with GC [32]. Sima et al. reported that higher levels of CCL5 in plasma or tissues might indicate an unfavorable prognosis in patients with GC [33]. Baj-Krzyworzeka et al. revealed that high serum levels of CCL5 were observed in GC cases [10]. Cao et al. showed that the expression of RANTES and its receptor, CCR5, can promote the development of metastasis and can be used as a marker of GC metastasis [34]. Ding et al. suggested that CCL5 has a critical role in mediating carcinogenesis, and CCL5 may be therapeutically targeted [35]. In light of this, as the first study evaluating the usage of CCR5 serum levels in terms of the diagnosis of GC, we found that CCR5 levels correlated with the diagnosis of GC (*p* < 0.001, r = −0.449), and median serum levels of CCR5 were higher in the GC group than the controls. CCR5 might be used as a biomarker in the early diagnosis of GC with 94.2% sensitivity and 42.5% specificity when the cut-off value is taken at 12.1 pg/mL. Higher CCR5 levels were also associated with worse survival times (40.26 ± 4.08 months vs. 27.98 ± 2.18 months, *p* < 0.05). We detected that CCL5 levels correlated with the diagnosis of GC (*p* = 0.014, r = −0.234). However, the difference between median CCL5 levels was not statistically significant (*p* = 0.141). Also, CCL5 levels did not affect survival (38.60 ± 3.99 months vs. 29.36 ± 2.39 months, *p* > 0.05). Except for CCL5 levels and their lack of association with survival, increased CCR5 levels and the correlation between CCL5/CCR5 levels and GC are consistent with the known literature-based data in compliance with a link between the CCL5/CCR5 axis and GC progression. These findings support the concept that levels of CCL5/CCR5 are valuable in the early diagnosis of GC. Although CCL5 and GC development were correlated, the fact that CCL5 levels were not statistically significant suggested that CCL5 levels were also high in the healthy control group. This may have been due to regional, environmental, or patient-specific factors.

The family of PDGF is a subgroup of tyrosine kinase receptors (TKR), which are critical actors in regulating diverse vital cellular processes such as growth, adhesion, differentiation, migration, and apoptosis [36]. The family of PDGF contains five dimeric isoforms: AA, BB, CC, DD, and the AB heterodimer [16]. The coupling of the PDGFs to their receptors on the cell membrane surface triggers expression in various cells and tissues [15]. Among various isoforms of PDGFs, PDGF-BB is directly implicated in the cell transformation process and the growth and progression of tumors [15,16]. Moreover, the effects of PDGF-BB on endothelial cell proliferation and cancer angiogenesis have also been demonstrated [37,38]. The positivity of PDGF-B (PDGF-AB/PDGF-BB) expression was higher in GC tissue than in non-pathological gastric mucosa [39,40]. Furthermore, PDGF-B expression correlated with the invasion potential of cancer and tumor stage progression [36]. The secretion of PDGF-B by gastric tumor tissue was shown to be associated with lymphatic spread [41]. As an emerging biomarker, studies have evaluated it to improve the accuracy of detecting PDGF-BB for early diagnosis [15,42]. The use of PDGF-BB as a biomarker for early diagnosis is suggested in esophageal cancer, prostate cancer, cholangiocarcinoma, and colorectal carcinoma [43,44,45,46]. However, there has been no study evaluating the role of PDGF-BB in the early diagnosis of GC. In this study, there was a correlation between PDGF-BB levels and the diagnosis of GC (*p* < 0.001, r = −0.700), and the median serum levels of PDGF-BB were higher in the GC group than in the controls. PDGF-BB may be a valuable biomarker in the early diagnosis of GC, with 91.3% sensitivity and 77.5% specificity when the cut-off value is taken at 59.8 ng/L. Higher PDGF-BB levels were also a significant parameter in predicting worse survival (45.24 ± 4.90 months vs. 27.59 ± 1.91 months, *p* < 0.05).

Eph receptors (erythropoietin-producing hepatocellular carcinoma), which constitute a large subgroup of the TKR family, play critical roles in interactions such as vascular development, cell migration and proliferation, and angiogenesis. Overexpression of the Eph receptors is an active player in carcinogenesis as they contain many oncogenes and proto-oncogenes. Therefore, they are promising drug targets [47]. EphA7 (Ephrin type-A receptor 7) is a member of this group, but there is still insufficient information in the literature about its association with cancer. *EphA7* gene mutations were also found more often in lung cancer specimens [19]. The *EphA7* expression was found in gastric cell lines and expressed in gastric carcinoma specimens. In several studies, the overexpression of EphA7 correlated with patient age and more advanced GC. The more frequent association of overexpression with young age has been interpreted as a role of EphA7 in gastric carcinogenesis from an early age [17]. We assessed the usage of EphA7 serum levels in terms of the diagnosis of GC, and we discovered that EphA7 levels correlated with the diagnosis of GC (*p* < 0.001, r = −0.617). The median serum levels of EphA7 were higher in the GC group than in the controls. EphA7 might be used as a biomarker in the early diagnosis of GC with 91.3% sensitivity and 67.5% specificity when the cut-off value is taken at 1.9 ng/mL. In addition, patients with high plasma EphA7 levels had reduced overall survival compared to those with low plasma EphA7 levels (40.48 ± 4.38 months vs. 29.42 ± 2.36 months, *p* < 0.05).

There are some limitations of the study. Because it was a single-center study, a sufficient number of subjects could not be included. Hence, due to the small number of subjects and the non-homogeneous distribution of tumor-specific parameters such as tumor grade or stage of disease, the results of these analyses may suggest conflicting conclusions. As a natural consequence of the time constraints included in this study, no objective assessment was performed on the predictive value of the parameters examined for the early detection of GC in healthy individuals. We need larger and multi-center studies to validate our findings.

## 5. Conclusions

As far as we know, this is the first study that has examined the potential connection between GC and serum levels of PDGF, EphA7, CCR5, and CCL5. As a result, the serum CCR5, EphA7, and especially PDGF-BB levels of the patients with GC were significantly higher than those in healthy individuals. Also, the outcomes obtained from the ROC analyses suggest that these molecule levels are valuable parameters with high accuracy in the early detection of GC. In addition, from this study, it may be extrapolated that CCR5, PDGF-BB, and EphA7 may be helpful parameters in predicting survival.

## Figures and Tables

**Table 1:** Characteristics of the patients and disease.

Variables	n
Number of patients	69
Age Mean ± SD:	61 ± 11
Sex Male/female	55/14
BMI Median (95% CI)	22 (21.6–23.9) kg/m^2^
Grade 1/2/3	1/13/47
Location Cardia/antrum/corpus	12/11/18
T stage 1/2/3	1/11/20
N stage 0/1/2/3	1/7/5/18
Stage II/III/IV	3/16/36

**Table 2:** The values of serum *CCR5*, *CCL5*, *PDGF-BB*, and *EPHA7* levels in patients with gastric cancer and healthy controls.

Assay	Patients (n = 69) Median (95% CI)	Controls (n = 40) Median (95%CI)	*p*
CCR5 (pg/mL)	64.8 (52.2–246.1)	24.1 (7.5–75.6)	<0.001
CCL5 (ng/L)	38.7 (28.7–65.8)	39.9 (16.9–54.6)	0.141
PDGF-BB (ng/L)	272.4 (145.7–812.9)	16.6 (12.5–53.2)	<0.001
EphA7 (ng/mL)	8.6 (5.8–44.5)	1.5 (0.9–1.9)	<0.001

**Table 3:** Results of the ROC analysis of *CCR5*, *CCL5*, *PDGF-BB*, and *EPHA7*.

Assay	Cut-Off Value	Sensitivity	Specificity	PPV	NPV %	AUC (CI) *
*CCR5* (pg/mL)	12.1	94.2%	42.5%	73.9%	81%	0.71 (0.61–0.81)
*CCL5* (ng/L)	15.6	87%	32.5%	69%	59.1%	0.58 (0.47–0.69)
*PDGF-BB* (ng/L)	59.8	91.3%	77.5%	87.5%	83.8%	0.92 (0.87–0.97)
*EphA7* (ng/mL)	1.9	91.3%	67.5%	82.9%	81.8%	0.83 (0.74–0.91)

* Asymptotic significance levels of *CCR5*, *CCL5*, *PDGF-BB*, and *EPHA7* were *p* < 0.001, *p* = 0.14, *p* < 0.001, and *p* < 0.001, respectively. AUC: area under the curve; CI: confidence interval; PPV: positive predictive value; NPV: negative predictive value.

## Data Availability

Not applicable.
